# Polycyclic Aromatic Hydrocarbons in Marine Environments Affect Fish Reproduction—A Critical Review

**DOI:** 10.3390/toxics13090747

**Published:** 2025-09-01

**Authors:** Roberta Pozzan, Aliciane de Almeida Roque, Hissashi Iwamoto, Fernando de Campos Guerreiro, Ana Paula da Silva, Dámaso Angel Rubio-Vargas, Micheli de Marchi, Felipe de Oliveira, Walter José Martínez-Burgos, Maritana Mela Prodocimo, Ciro Alberto de Oliveira Ribeiro

**Affiliations:** 1 Cell Toxicology Laboratory, Department of Cell Biology, Biological Sciences Sector, Federal University of Paraná, Curitiba CEP 81531-970, PR, Brazil; alicianeroque@gmail.com (A.d.A.R.); fernandocguerreiro@outlook.com (F.d.C.G.); paulaana@ufpr.br (A.P.d.S.); cubanum@gmail.com (D.A.R.-V.); michelidemarchi27@gmail.com (M.d.M.); felipe.de@ufpr.br (F.d.O.); maritana.mela@gmail.com (M.M.P.); ciro@ufpr.br (C.A.d.O.R.); 2 Department of Bioprocess Engineering and Biotechnology, Federal University of Paraná, Curitiba CEP 82590-300, PR, Brazil; hissashi.iwamoto@gmail.com

**Keywords:** polycyclic aromatic hydrocarbons, endocrine disruption, fish reproduction, marine pollution, marine biomonitoring

## Abstract

The biodiversity of marine and coastal ecosystems is constantly threatened by pollutants from a diversity of human activities. The polycyclic aromatic hydrocarbons (PAHs) are a class of pollutants widely released and deposited in these environments, leading to several impacts on the community of organisms that integrate these ecosystems. As lipophilic compounds, PAHs become bioavailable to organisms and can enter the trophic chain, leading to physiological changes and affecting different levels of biological organization. Several studies demonstrate that PAHs act as endocrine disruptors in marine fish, interfering with endocrine signaling through hormonal disturbances and, consequently, causing inhibition or overexpression of genes, enzymes, and proteins that are essential for reproduction success. These changes, in turn, can lead to population decline and cause immeasurable ecosystem damage. This review synthesizes studies published mainly between 2015 and 2025, aiming to critically present research that identifies different endocrine-reproductive changes in marine fish species exposed to PAHs in contaminated sites, highlighting the involved cellular mechanisms. Finally, we provide a survey of patents developed to identify PAHs in aquatic environments and how these techniques can be used in marine biomonitoring to evaluate water quality and the risk of exposure to biota and human populations.

## 1. Introduction

Polycyclic aromatic hydrocarbons (PAHs) are a diverse group of organic contaminants originating from both natural and anthropogenic sources, including oil extraction, industrial activities, vehicular emissions, and fossil fuel burning [1]. They are widely distributed in marine environments and recognized as indicators of environmental contamination, particularly in areas impacted by oil spills and urban runoff [2].

Due to their hydrophobic nature, PAHs partition to suspended particles and accumulate in sediments, where their persistence depends on compound properties and environmental conditions [3]. In marine ecosystems, high salinity, low temperatures, and anoxic zones contribute to limited microbial activity and enhanced sorption, resulting in half-lives of years to decades [4]. In contrast, freshwater ecosystems, with lower ionic strength, more oxygenated sediments, and a more diverse and active microbial community, tend to promote faster PAH degradation [5].

Temporary accumulation can occur in lipid-rich tissues of vertebrates or in species with limited detoxification capacity, such as invertebrates [6,7]. In fish, exposure to PAHs has been associated with genotoxicity, neoplastic lesions, and disturbances in energy metabolism [8,9]. Notably, many PAHs exhibit endocrine-disrupting properties, interfering with hormonal regulation and reproductive processes [10]. Early developmental stages are especially vulnerable, given their high growth rates and underdeveloped detoxification systems [11,12].

This review critically examines the knowledge on the reproductive toxicity of PAHs in marine fish species through a comprehensive search of peer-reviewed literature available in databases including Web of Science, Scopus, and PubMed. Articles published between 2015 and 2025 were prioritized in order to highlight recent advances, although earlier seminal studies were also considered when relevant to contextualize mechanisms. Studies were included if they explored the effects of PAHs on reproductive endpoints in fish, with particular emphasis on marine species. Freshwater fish studies were included selectively to provide mechanistic insights when data for marine organisms were scarce.

## 2. PAHs in Aquatic Environments

PAHs are structurally characterized by multiple aromatic rings and are known for their toxicity, mutagenicity, genotoxicity, and carcinogenicity [13,14]. Over 100 PAHs have been identified, occurring individually or as mixtures [15], classified as high molecular weight (HMW, ≥4 rings) or low molecular weight (LMW, ≤3 rings) [16]. Their sources include natural ecological processes (wildfires, volcanic activity) or anthropogenic activities (oil extraction, industrial activities, vehicular emissions, fossil fuel burning) [17], and they can be found as components of crude oil and its derivatives (asphalt, gasoline, diesel). The widespread use of these compounds has resulted in their pervasive presence in the environment, particularly within aquatic ecosystems [18].

These compounds exhibit low to extremely low water solubility and vapor pressures ranging from low to moderately high [19,20]. These characteristics, combined with their widespread emissions, make them prevalent in water and sediments [21]. In marine environments, PAHs undergo partial volatilization and dissolution in seawater, while a significant fraction rapidly adsorbs onto suspended particles and sediments or becomes associated with petroleum-derived fractions [22]. Atmospheric deposition also contributes to PAH inputs in the oceans [23], and ocean currents facilitate their dispersion [24,25].

PAHs have been detected in different marine ecosystem stratification, from surface waters to sediments [2]. Concentrations vary according to anthropogenic activity: for example, 1 to 251 ng/g dry weight (dw) in Moroccan Mediterranean sediments [26], 3.15 to 14.35 ng/g (dw) in Qatari coastal sediments [27], and 236.27 to 644.87 ng/g (dw) in the Brazilian Suape Port sediments, with highest levels near shipyards [28]. Such findings demonstrate their widespread distribution and persistent, reinforcing the need for continuous monitoring and remediation [14].

## 3. Uptake and Transient Accumulation of PAHs in Marine Organisms

The uptake of PAHs in marine organisms refers to the process by which these hydrophobic organic compounds enter biological tissues through water, sediments, or diet [29]. PAH absorption mechanisms in fish include direct diffusion through gills and skin, as well as ingestion of contaminated prey or particles [30,31,32].

Once absorbed, PAHs are distributed via the bloodstream, demonstrating high affinity for lipid-rich tissues, such as the liver, where they undergo biotransformation [33]. Nevertheless, transient accumulation can occur in some tissues, where higher concentrations may be detected shortly after exposure [30]. In this context, bile is another sensitive pathway, especially for evaluating recent PAH exposure, due to its role in their metabolite excretion [34,35,36].

Due to their direct contact with water, gills also accumulate PAHs, as shown in several marine fish species [32,37]. In contrast, muscle tissue typically accumulates lower concentrations of PAHs compared to lipid-rich tissues. However, its relevance increases due to its role in human consumption [38]. Other tissues, such as the brain, kidneys, and skin, may also accumulate PAHs, though studies on these tissues focus primarily on structural, physiological, or biochemical effects [35,39,40,41].

The composition of PAH mixtures in ecosystems also plays a significant role: high molecular weight (HMW) compounds tend to accumulate more in organisms, likely due to a correlation between their trophic magnification factors (TMF) and log *K*_ow_ values, as well as the generally lower metabolic capacity of organisms to biotransform larger PAHs [42,43].

The bioavailability of PAHs is influenced by their total concentrations in sediments and by the fraction that is chemically active, that is, the freely dissolved and desorbable portion of PAHs that can equilibrate between sediment particles, porewater, and organisms [44]. Furthermore, physiological traits of species, such as metabolic rates, lipid storage capacity, and diet composition, significantly modulate accumulation [45,46].

In marine trophic chains, PAH concentrations can vary significantly due to interactions between prey and predators. These variations are influenced by opposing processes: biomagnification, where contaminant concentrations increase with each trophic level, and biodilution, in which concentrations decrease due to dilution effects [42]. Some studies suggest that biomagnification is dominant, particularly in predator species with high lipid content [45,47]. However, recent research highlights biodilution as a relevant mechanism in marine trophic networks, since LMW PAHs exhibit lower log *K*_ow_ values and TMFs < 1, reducing their accumulation in higher trophic levels [43,48].

In longer and more lipid-rich food webs, biomagnification tends to prevail, whereas in systems dominated by species with high metabolic capacity or shorter trophic chains, biodilution is more likely [42]. This indicates that the trophic transfer dynamics of PAHs are highly complex, being influenced by both ecological characteristics and the physicochemical properties of these compounds.

## 4. Impacts of PAHs on Marine Fish: Bioindicator Responses and Biological Effects

Pollution by PAHs in marine ecosystems poses a significant threat to biota, especially fish, which play crucial roles in food chains [49]. The use of fish as bioindicator organisms is widely known, due to their sensitivity to environmental changes and prolonged exposure to toxic compounds [50,51,52,53,54]. Several biomarkers have been widely used to monitor the impacts of PAHs in fish, providing early information on exposure levels and toxicity [55,56,57]. Table 1 presents a summary of different effects caused by exposure to PAHs in marine fish species, providing an overview of the consequences on different species under PAH exposure, highlighting the relevance of fish as sentinel organisms in marine environments.

At early developmental stages, PAHs can disrupt embryogenesis and larval survival in marine fish. Cardiac malformations (pericardial edema, arrhythmias, and dysfunction) are common. Craniofacial anomalies, fin deformities, delayed hatching, and impaired swim bladder inflation can compromise juvenile recruitment and energy efficiency [29,41,69,71,72,73,74].

In adult marine fish, PAHs also impair cellular homeostasis and organ integrity. Phenanthrene disrupts intracellular calcium cycling, impairing cardiac contractility and muscle development [69,73], while alkylated PAHs such as acenaphthene suppress osteoblastic activity, leading to skeletal deformities and structural fragility [29]. HMW PAHs (e.g., BaP) bind to the aryl hydrocarbon receptor (AhR), activating xenobiotic metabolism pathways and generating reactive intermediates that induce oxidative stress and DNA damage, often culminating in carcinogenesis [29,73]. Additional effects include metabolic disruption of lipid and glucose pathways [29], ocular degeneration [75], neurotoxicity, oxidative damage [76,77,78], and immune suppression through phagocytosis, lymphocyte proliferation [79], and cytokine imbalance [80,81].

These physiological and molecular disruptions can compromise both individual and population viability. Cardiac and swim bladder impairments reduce swimming efficiency, limiting predator evasion, foraging, and migration [69]. Ocular degeneration and metabolic disruptions could explain lethargic behaviors and anxiety-like responses [70,82]. Furthermore, delayed juvenile growth reduces competitive advantage and survival, and delays sexual maturation, ultimately affecting population replenishment [71,72]. Such disruptions can also cause intergenerational effects, as epigenetic changes may perpetuate population decline. Consequently, the decrease in forage fish leads to trophic alterations, destabilizing food webs [41] and resulting in population collapses [69].

## 5. Impacts of PAHs on Marine Fish Reproduction

Reproduction is one of the biological processes most sensitive to interference from environmental contaminants, especially endocrine-disrupting compounds such as PAHs. In marine fish, reproduction depends on a complex network of hormonal, molecular, and physiological interactions that coordinate everything from sexual differentiation and gamete production to behaviors associated with spawning and parental care [83]. Any dysfunction in this system can compromise not only individual reproductive performance but also population viability in the medium and long term [84].

Several studies have shown that PAHs are capable of interfering at multiple levels of fish reproductive physiology, from hormonal signaling in the central nervous system to histological changes in the gonads [85]. These interferences can occur through different mechanisms, including hormonal mimicry, inhibition of steroidogenic enzymes, modulation of nuclear receptors, oxidative stress, and epigenetic changes. Furthermore, reproductive responses to PAH exposure often do not follow linear dose-response patterns and may vary according to species, sex, developmental stage, and duration of exposure [41].

This section critically addresses the main reproductive effects of PAHs in marine fish, based on recent and relevant evidence from the scientific literature. Changes in key genes, proteins, processes, and structures involved in marine fish reproduction are discussed. This integrated approach seeks to highlight not only the isolated effects of PAHs but also their systemic and ecological implications on marine reproduction in the face of PAH contamination.

To synthesize the current scientific evidence on PAH-induced reproductive effects in marine fish, Table 2 presents a structured summary of experimental and field studies published in the last ten years. This compilation includes details of the literature references and provides an integrative overview of how various PAH compounds affect marine fish reproduction across different biological and ecological contexts.

### 5.1. Metabolism of PAHs, Induction of Cytochrome P450 Enzymes, and Reproduction

The biotransformation of PAHs in marine organisms is a process largely mediated by enzymes of the cytochrome P450 family, notably CYP1A, whose expression is regulated by the activation of the aryl hydrocarbon receptor (AhR) after the pollutant enters the cell [104,113]. This mechanism has been consistently identified as one of the main detoxification pathways, but it is not entirely risk-free, since the metabolites formed are often more toxic and reactive than the original compounds [29,114]. In addition, the metabolic activation of PAHs through CYP1A can generate reactive oxygen species (ROS), which contribute to oxidative stress and further exacerbate cellular and reproductive damage [29].

**Figure 1:**
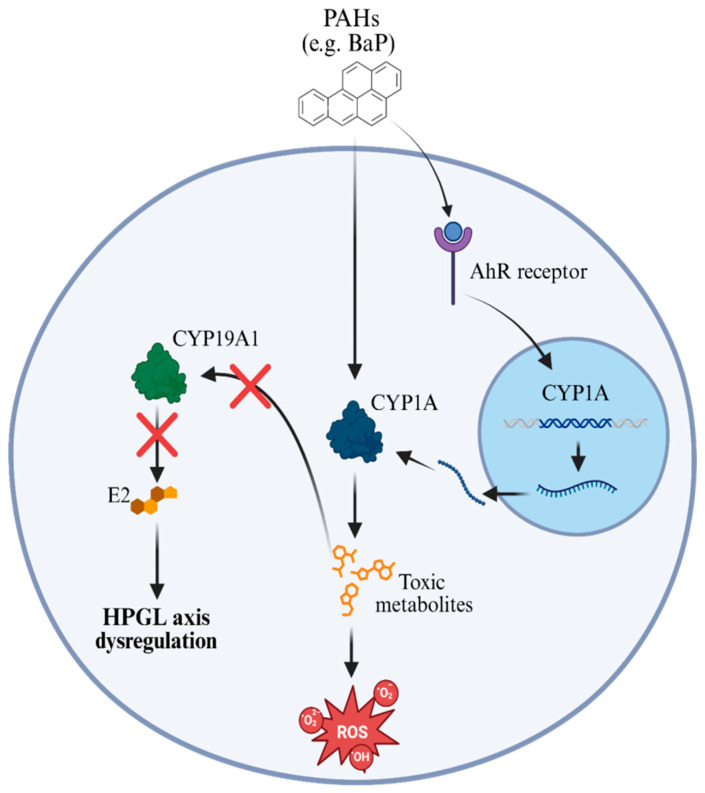
AhR-CYP1A pathway induced by PAHs and interference in the HPGL axis. After crossing the cell membrane, PAHs bind to the aromatic hydrocarbon receptor (AhR), activating the transcription of the *Cyp1a* gene in the nucleus. The CYP1A enzyme metabolizes PAHs into reactive and toxic compounds, which promote the generation of reactive oxygen species (ROS), leading to oxidative stress and cellular damage. In parallel, these metabolites negatively interfere with the expression and function of aromatase (CYP19A1), the enzyme responsible for the conversion of androgens into 17β-estradiol (E2). The reduction in E2 synthesis compromises hormonal signaling and results in dysregulation of the hypothalamic-pituitary-gonad-lobe (HPGL) axis, negatively affecting the reproductive function of exposed fish.

Aromatase, an enzyme encoded by the *Cyp19* gene and responsible for the conversion of androgens into estrogens, plays a central role in the regulation of reproduction in fish. In *Liza klunzingeri*, cellular exposure to benzo[a]pyrene (BaP) was shown to significantly inhibit aromatase activity in both ovarian and brain cells, consequently reducing 17β-estradiol (E2) levels. This inhibition, observed in a dose-dependent manner, highlights the antiestrogenic potential of BaP, an endocrine disruptor that interferes with steroid homeostasis through mechanisms mediated by AhR [92]. Such mechanisms include direct suppression of *Cyp19a1* gene transcription, in addition to conversion of estrogens into less active forms, impairing estrogen signaling. Reduced aromatase activity compromises estrogen biosynthesis and may negatively affect the hypothalamic-pituitary-gonadal (HPG) axis, impacting fertility and reproductive function of fish exposed to PAHs such as BaP [115]. These results highlight aromatase as a sensitive target of PAH contamination and a promising biomarker of environmental endocrine dysfunction, as shown in Figure 1.

Studies using precision-cut liver slices (PCLS) of cod species have further expanded the understanding of PAH antiestrogenic effects and its interaction with estrogen signaling. In *Gadus morhua*, exposure to BaP, EE2, and their equimolar mixtures for 48 h revealed distinct transcriptional profiles: BaP strongly induced AhR-related genes such as CYP1a and AHRr, whereas EE2 upregulated classic estrogen-responsive genes such as VTG and ZP. However, co-exposure resulted in attenuation of the estrogenic response, indicating functional antagonism by BaP [97]. A complementary study in *Boreogadus saida* simulated early vitellogenic conditions using physiological concentrations of EE2 (5 nM) and prolonged exposure (72 h) and demonstrated dose-dependent suppression of ESR1 and VTG1 expression by BaP. Transcriptomic analyses suggested that these antiestrogenic effects may involve multiple molecular mechanisms, including estrogen receptor degradation, interference with promoter binding, competition for coactivators, and depletion of endogenous estrogens [97]. Together, these findings confirm that AhR activation by BaP not only drives xenobiotic metabolism through CYP1 induction but also disrupts estrogen signaling pathways, posing a risk to reproductive fitness in fish chronically exposed to PAH mixtures.

Chronic in vivo exposure studies have also demonstrated the biological relevance of CYP1A induction in environmentally realistic scenarios [102,116,117,118,119]. Juvenile southern flounder (*Paralichthys lethostigma*) exposed for 32 days under controlled laboratory conditions to five concentrations of weathered Macondo MC252 oil mixed with field-collected sediments exhibited a dose-dependent upregulation of CYP1A expression in liver tissue. In these exposure sediments contained between 0.04 and 395 mg/kg of total PAHs (tPAH_50_), and fish exposed to concentrations above 8 mg/kg showed significantly reduced growth and increased mortality. These findings emphasize that PAH-induced molecular responses, such as CYP1A induction, are not only sensitive biomarkers of exposure but also correlate with significant physiological impairments in fish health [90].

The induction of the *Cyp1a* gene is also commonly monitored through the activity of the enzyme EROD (ethoxyresorufin-O-deethylase), which is widely considered a reliable biomarker of PAH exposure in fish [105,120]. In *Sebastes schlegelii* (black rockfish), exposure to benzo[a]pyrene (BaP) at concentrations of 2, 20, and 200 μg/g body weight resulted in a dose-dependent increase in both hepatic EROD activity and microsomal CYP1A protein levels. Notably, at 200 μg/g, BaP induced a four-fold increase in EROD activity and a six-fold increase in CYP1A protein expression [96]. In studies with polar cod (*Boreogadus saida*), prolonged exposure to crude oil also demonstrated a gradual increase in EROD activity, evidencing a cumulative effect of environmental exposure [101].

However, it is important to highlight that, although the increase in CYP1A is useful as a marker of exposure, the correlation with adverse physiological effects is not always linear [12,103,121]. As evidenced by [99,122], AhR activation can occur with different potencies depending on the composition of the PAH mixture, the species, and the sex, generating variable responses even under similar exposure conditions.

Furthermore, interference by endogenous estrogens on CYP1A activity may significantly affect detoxification and steroid regulation mechanisms, especially during critical reproductive periods, as observed by [123]. CYP1A suppression by estrogens during sexual maturation, for example, may represent an adaptive response to protect hormone levels necessary for reproduction but may also increase vulnerability to toxicity from unmetabolized PAHs [106].

The discrepancy between enzyme activation and effective toxicity suggests that biomarkers such as CYP1A and EROD, while informative, should not be interpreted in isolation. A comprehensive assessment requires their integration with physiological, reproductive, and genomic analyses to accurately elucidate the real impact of chronic exposure to PAHs on the reproductive biology of marine fish [124,125].

### 5.2. Disruption of the Hypothalamic-Pituitary-Gonad-Lobe (HPGL) Axis

The functional integrity of the hypothalamic-pituitary-gonad-lobe (HPGL) axis is essential for the regulation of reproduction in marine fish, coordinating hormonal synthesis from the central nervous system to the final maturation of gametes [126]. Exposure to PAHs has been shown to be a significant disruption factor in this axis, resulting in hormonal, epigenetic, and histological changes that seriously compromise the reproductive success of exposed species [127].

Studies with marine medaka (*Oryzias melastigma*) chronically exposed to phenanthrene (PHE) and PHE-adsorbed microplastics have demonstrated bioaccumulation of the contaminant in the brain and ovaries, accompanied by reduced expression of key genes in the HPGL axis, such as GnRH, LHβ, FSHβ, and CYP19a [91]. These changes are directly associated with decreased synthesis of gonadotropins (LH and FSH), compromising steroidogenesis and, consequently, the production of sex hormones such as estradiol (E2) and testosterone (T). This condition translates clinically into follicular atresia, inhibited ovarian maturation, and lower egg production, with potentially transgenerational effects [115].

In addition to the direct modulation of HPGL axis genes, there is evidence that PAHs interfere with hormonal feedback mechanisms that regulate hypothalamic and pituitary function. Ref. [89] demonstrated that cod exposed to mixtures of PAHs showed reduced GnRH2 expression and a concomitant increase in dopaminergic receptors (DRD2a) in the brain, indicating that the dopaminergic pathway is particularly sensitive to exposure to PAHs. Since dopamine negatively regulates GnRH secretion, these neural changes can cause significant reductions in pituitary stimulation, resulting in downstream gonadal dysfunction [115].

An additional and critical layer of disruption is mediated through epigenetic mechanisms. Studies have shown that embryonic exposure to water-soluble fractions of petroleum (WAF) increases methylation of the VTG gene, associated with decreased expression of GnRH, LHβ, FSHβ, and CYP19b, with deleterious consequences for ovarian maturation and hormonal balance [22]. This form of epigenetic modification is particularly concerning, as it may lead to latent or heritable effects, affecting populations across generations even after the end of direct exposure.

Ref. [87] observed that PHE exposure caused a U-shaped dose-response curve in the mRNA levels of GnRH, FSH, and LH in male fish, a phenomenon known as hormesis. The interpretation of this pattern is complex, but it points to non-linear neuroendocrine dysfunctions, challenging the traditional idea of dose-dependence and making it difficult to predict the population effects of PAH contamination.

The impact on HPGL is even more evident in species with sexual plasticity, such as *Epinephelus marginatus*, a protogynous hermaphrodite. Ref. [86] observed that exposure to PHE caused reductions in the levels of 11-ketotestosterone (11-KT) in this fish, essential for sexual inversion, in addition to dysfunctions in steroidogenesis, directly affecting reproductive development and population dynamics.

**Figure 2:**
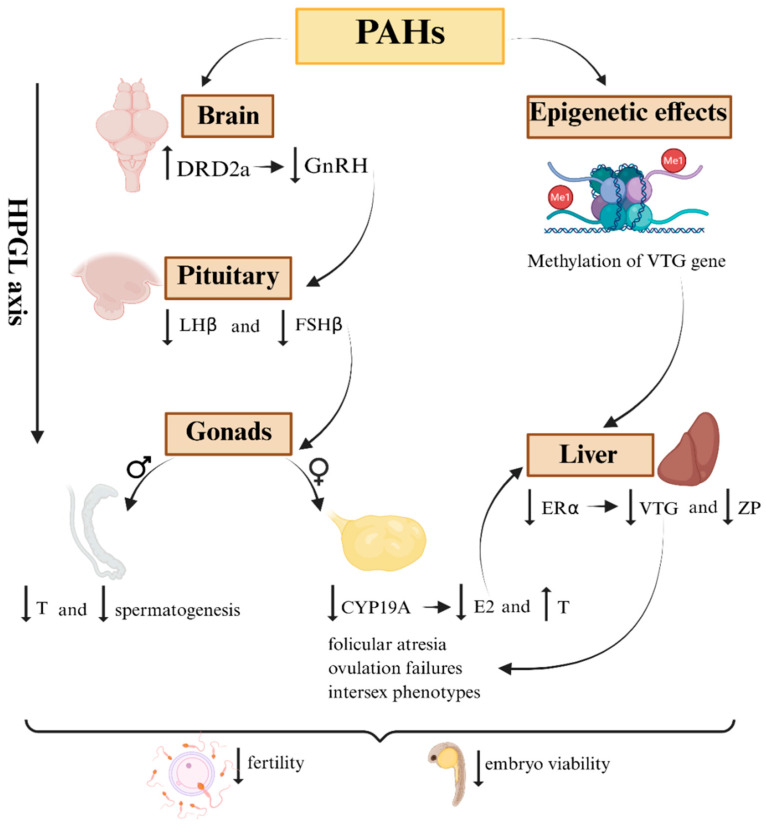
Schematic representation of how PAHs disrupt the HPGL axis in marine fish. In the brain, an increase in the expression of dopaminergic receptors (DRD2) inhibits the release of gonadotropin-releasing hormone (GnRH). This dysregulation reduces the hypothalamic stimulus to the pituitary gland, resulting in a decrease in the expression and release of the gonadotropins LHβ and FSHβ. Subsequently, the effects extend to the gonads, where aromatase (CYP19a) is suppressed. As a consequence, there is an accumulation of testosterone (T) and a reduction in estradiol levels (E2). This leads to a reduction in the expression of estrogen receptors (ERα), vitellogenin (VTG), and zona pellucida proteins (ZP2 and ZP3) in the liver. Epigenetics represents an additional axis of dysregulation, with increased methylation in key genes such as VTG, resulting in persistent or transgenerational changes even after the end of exposure.

Therefore, the available evidence suggests that PAHs interfere at multiple levels of the HPGL axis, ranging from the expression of GnRH in the hypothalamus to gametogenesis. This interference does not always manifest itself in a time-, dose-, and compound mixture-dependent manner, in addition to being modulated by neuroendocrine and epigenetic interactions. These findings reveal that HPGL is not only one of the main targets of PAH toxicity but also a possible link between individual alterations and population collapses in marine fish exposed to these pollutants [128]. These multilevel interferences along the HPGL axis are illustrated in Figure 2, which summarizes the main neuroendocrine and epigenetic pathways through which PAHs disrupt fish reproduction.

### 5.3. Changes in Reproductive Hormones

Exposure to PAHs has been widely associated with changes in sex hormone levels in marine fish, affecting both the synthesis and regulation of reproductive steroids such as 17β-estradiol (E2), testosterone (T), 11-ketotestosterone (11-KT), LH, FSH, and maturation-inducing hormones. These hormonal changes underlie several reproductive dysfunctions observed in fish exposed to PAHs in contaminated environments, indicating a profound interference in the reproductive neuroendocrine axis [129].

Several studies have shown that PAHs can significantly reduce circulating levels of estradiol and testosterone, with direct implications for gonadal development and fertility [10]. Disruption in the biosynthesis of these sex hormones compromises the natural reproductive cycle and may interfere with the population dynamics of several species [130]. For example, studies with marine fish exposed to PAHs indicated consistent reductions in plasma E2 levels and parallel increases in T levels, altering the balance between estrogens and androgens and resulting in follicular atresia, reduced egg production, and decreased oocyte quality [91]. Elevated testosterone levels, in addition to indicating a failure in aromatization (conversion of T to E2), may also exert inhibitory effects on ovarian maturation through negative feedback mechanisms, aggravating hormonal dysfunction [131].

Another critical aspect observed refers to dysfunction in the expression and regulation of gonadotropic hormones, such as FSH and LH. Ref. [87] reported that exposure to PAHs led to downregulation of the LHβ and FSHβ genes, with effects observed on both transcription and hormone release, which compromises ovarian follicle maturation and gamete production. These changes can occur even at environmentally relevant levels of PAHs, suggesting high sensitivity of the pituitary axis to these compounds [132].

Interestingly, Ref. [22] observed that embryonic exposure to WAF significantly altered the expression levels of GnRH, LHβ, and FSHβ in adulthood, indicating an early endocrine programming effect with late consequences on hormonal regulation. This finding reinforces the hypothesis that PAHs can generate latent reproductive effects, including transgenerational ones, through interference in the HPGL axis during embryonic development [133].

Although the most frequently reported effects involve the suppression of female hormones such as E2, there are also records of paradoxical increases in this hormone in some species and conditions. In juvenile female cod exposed to low doses of PAHs, an increase in plasma E2 levels was observed [89]. This nonlinear response is attributed to the complex interaction between PAH metabolization pathways, AhR activation, and hormone receptors, in addition to the modulation of dopaminergic pathways that participate in the control of GnRH and, consequently, the release of gonadotropins.

This diversity of responses demonstrates that the effects of PAHs on reproductive hormones do not conform to a straightforward dose-response relationship. Instead, they are often modulated by factors such as species, sex, developmental stage, route of exposure, mixture of compounds, and duration of contact. Furthermore, interference in the expression of steroidogenic enzymes, such as CYP17 and CYP19, further exacerbates hormonal imbalances, indicating that the metabolism of sex steroids is one of the main targets of PAH toxicity [130].

Finally, it is important to highlight that hormonal effects do not occur in isolation but are integrated into a cascade of physiological and behavioral events that culminate in reproductive dysfunction. The reduction of sex hormones compromises the development of gonads, gamete production, embryo viability, and even behaviors associated with copulation and spawning, rendering PAHs particularly detrimental to the reproductive success of marine fish in ecosystems impacted by oil pollution [100].

### 5.4. Estrogenic and Anti-Estrogenic Effects

The estrogenic and antiestrogenic effects of PAHs represent one of the most complex aspects of endocrine disruption induced by these compounds in marine fish. Several PAHs and their hydroxylated metabolites have chemical structures similar to that of 17β-estradiol (E2), enabling them to bind to estrogen receptors (ERs). Depending on the biological context, target tissue, and dose, these compounds may act as either agonists or antagonists, thereby modulating estrogenic signaling in diverse and sometimes unpredictable ways [29].

This dual nature of PAHs poses a challenge for predicting their reproductive effects and for environmental toxicological modeling. Recent literature highlights that many PAHs, especially their hydroxylated derivatives such as 9-OH-benzo[a]pyrene and 1-hydroxynaphthalene, activate ERs in vitro, promoting gene transcription and expression of proteins typical of estrogenic action, such as vitellogenin [134]. On the other hand, other PAHs act as ER antagonists, blocking the action of estradiol and reducing the expression of estrogen-sensitive genes, as observed in the decrease in VTG and ERα in the liver and ovaries of *Solea solea* females exposed to benzo[a]pyrene [88].

The duality in the effects of PAHs is also reflected in the different types of estrogen receptors expressed in the target tissues. Even under conditions of reduced E2, there was a compensatory increase in the expression of ERα and ERβ in the liver and ovaries of marine medaka exposed to WAF. This overexpression can be interpreted as a cellular attempt to restore estrogen signaling in the face of hormonal deficiency, but it can also aggravate tissue sensitivity to xenoestrogenic compounds, amplifying toxicity in scenarios of prolonged exposure [22].

The inconsistency between plasma E2 levels and tissue expression of ERs and target genes also points to the existence of regulatory pathways independent of endogenous estrogen. Ref. [89] demonstrated that, although exposure to low doses of PAHs increased plasma E2 levels in female cod, there was a significant reduction in the brain expression of ERα and aromatase CYP19a1b, suggesting that dopaminergic or epigenetic mechanisms could be interfering with brain estrogen sensitivity. This finding reinforces the idea that the estrogenic activity of a compound cannot be inferred solely based on circulating hormone levels [89].

Consistently, inhibition of aromatase (CYP19a) has been reported in several studies, reducing the conversion of T into E2 and promoting hormonal imbalances that impair ovulogenesis, alter reproductive behavior, and decrease fecundity [22,135]. In addition, the action of PAHs on ERs may be affected by the coactivation of alternative intracellular pathways, such as the aryl hydrocarbon receptor (AhR) [122]. Simultaneous activation of AhRs and ERs can lead to cross-talk responses, such as competition for transcription cofactors and mutual repression of gene expression, which contributes to the variability of the estrogenic effects of PAHs [122].

PAHs exert highly contextual estrogenic and antiestrogenic effects, mediated by interactions with ERs, aromatase inhibition, AhR activation, epigenetic alterations, and modulation of dopaminergic pathways. These multiple routes of action make the effects difficult to predict and indicate that PAHs can profoundly dysregulate estrogenic signaling in marine fish, with severe consequences for reproduction, especially in species with hormonal plasticity or estrogen-dependent development [136].

#### Production and Expression of Vitellogenin and Vitellin

Vitellogenin (VTG), a phosphoprotein precursor of vitellin, is one of the most sensitive and widely used biomarkers for monitoring endocrine disruption in fish, especially in the context of exposure to estrogenic and antiestrogenic contaminants such as PAHs [98,137]. VTG synthesis is induced by estrogens, particularly 17β-estradiol (E2), and occurs predominantly in the liver of maturing females. Following its secretion into the bloodstream, VTG is incorporated into developing oocytes, where it is proteolytically cleaved into various yolk proteins (e.g., vitellin, lipovitellin, phosvitin) that serve as key nutrient sources during embryogenesis. Any dysfunction in this process severely compromises reproduction [138,139].

Exposure to PAHs has been shown to exert bidirectional effects on VTG expression: aberrant induction of VTG in males and juveniles, evidence of exogenous estrogenic action, and/or suppression of VTG expression in females, reflecting antiestrogenic effects that impair ovarian function [130].

The study by [95] demonstrated a marked and recurrent induction of *vtg* gene expression in male flatfish from different regions of the Gulf of Mexico, strongly associated with the presence of PAHs in tissues and biliary metabolites. VTG expression was detected in up to 47.69% of males in some oceanographic campaigns, with levels comparable to or exceeding those observed in females, thereby indicating substantial hormonal dysregulation. PAHs were consistently correlated with this VTG expression, particularly the metabolites hydroxynaphthalene and hydroxyphenanthrene, which may substantially compromise the reproductive function of affected individuals. The persistent induction of VTG in males represents a warning sign for diffuse contamination by PAHs in the marine environment and its possible ecological repercussions [95].

In polar cod exposed to water-soluble fractions of crude oil, a significant decrease in the expression of hepatic genes associated with vitellogenesis, such as VTG α, ESR1, ZP2, and ZP3, all positively regulated by estrogens and typically expressed at high levels during the vitellogenesis phase of the reproductive cycle, was observed. The reduction of these genes suggests an antiestrogenic effect of oil exposure, potentially responsible for earlier spawning in exposed females, also observed in the study. Despite a later increase in VTG α expression after 131 days of exposure, this delayed response may reflect changes in the PAH composition of the oil mixture over time, which influence estrogen receptors differently depending on the concentration and type of bioavailable PAHs [100].

In addition to gene regulation, changes in hormonal signaling also explain the reduction in VTG. Fish exposed to PAHs showed a significant decrease in ER-α levels in the liver and ovaries, which probably contributed to the decrease in hepatic VTG production. Considering that estrogen receptor activation is a necessary step for VTG induction, the downregulation of ERs represents an important antiestrogenic mechanism [88].

Another important consideration is the distinction between VTG expression and its functional activity. Even when VTG is induced, as observed following exposure to PAH metabolites with estrogenic properties, this induction does not necessarily ensure that the resulting protein is functionally effective. Oocyte quality may be compromised, and elevated VTG levels in males or juveniles are often associated with the occurrence of intersex testes or infertility [140]. Therefore, the presence of VTG outside the expected physiological context is a marker of endocrine dysfunction. From an ecological perspective, the sustained reduction of VTG expression in females may result in reproductive failure at the population level, posing a significant threat to the long-term viability of affected fish communities. Persistently low levels of VTG are associated with reduced oocyte number and quality, lower hatching rate, and decline in the recruitment of new generations. This makes VTG not only a biomarker of exposure but also a functional indicator of reproductive success in impacted environments [141].

Also, it is important to consider the indirect effects of PAHs on VTG production, such as those mediated by alterations in CYP19a (aromatase), responsible for the conversion of testosterone (T) to estradiol (E2). The suppression of CYP19a, observed in several studies [87,91], reduces the levels of E2 available to stimulate VTG production, creating a cascade effect that compromises the entire vitellogenesis process.

### 5.5. Androgenic and Anti-Androgenic Effects

The action of PAHs on the androgenic pathway in marine fish has been less explored compared to their estrogenic effects, but recent studies reveal that these contaminants also exert important androgenic and anti-androgenic effects, with critical implications for sexual differentiation, gonadal maturation, and reproductive success, once PAHs can interfere with the synthesis, metabolism, signaling, and gene expression of androgens such as testosterone (T) and 11-ketotestosterone (11-KT) [142].

One of the main routes of toxicity involves the modulation of aromatase (CYP19), because of its role in converting T to estradiol (E2). When the expression of CYP19a is suppressed, androgens accumulate, with consequent hormonal imbalance. It indicates that the androgenic toxicity of PAHs can manifest itself both by androgen deficiency (antiandrogenic effect) and by secondary accumulation of T due to failure in conversion to E2 (indirect androgenic effect) [143].

At the molecular level, the expression of androgen receptors (arα and arβ) in fish exposed to PAHs may increase. This overexpression may be a compensatory attempt to the reduction of bioavailable T or an adaptive response to high levels of circulating T [142]. However, excessive activation of androgen receptors in female tissues can lead to partial masculinization, in addition to morphological and functional alterations in the gonads [22].

Previous studies also demonstrated that exposure to BaP reduced the expression of CYP19a and ERα, while increasing the expression of androgen receptors in the testes and liver of medaka, promoting a favored androgenic profile [94]. These changes were accompanied by changes in sex ratio, with a greater number of males in the offspring, suggesting that BaP may act as a masculinizing agent, including at embryonic stages. This may be particularly harmful for species with sensitive environmental or physiological sexing, as sex imbalances compromise population recruitment [144].

In addition, there is evidence that PAHs modulate androgen signaling indirectly, through activation of the AhR, which may interfere with steroidogenic pathways. This interaction was suggested by [89], who observed changes in the expression of dopaminergic and estrogenic genes after exposure to PAHs, but also changes in the expression of CYP19a1b, an enzyme that integrates the central control of estrogen production in the brain and influences the HPG axis. Thus, central aromatase dysregulation may also have an indirect impact on androgen homeostasis.

It is also important to note that the androgenic and antiandrogenic effects of PAHs, as well as estrogens, often follow non-monotonic curves and non-linear dose-dependent responses, which makes it difficult to predict population effects from laboratory studies with single or limited doses [145].

### 5.6. Thyroid Hormone Disruption and Its Effects on Reproduction

Although the effects of PAHs on the reproductive axis are widely recognized, recent studies have shown that these compounds also significantly interfere with the homeostasis of thyroid hormones (THs), directly and indirectly impacting the reproduction of marine fish [146]. Thyroid hormones, especially thyroxine (T4) and triiodothyronine (T3), are essential for the development, metabolism, and regulation of several physiological processes, including reproduction, metamorphosis, and sexual differentiation [112].

The synthesis and activation of THs occur through the hypothalamic-pituitary-thyroid (HPT) axis, with TSH (thyroid-stimulating hormone) being the central regulator of T4 production in the thyroid gland [147]. The peripheral conversion of T4 to T3, which is more biologically active, depends on the activity of hepatic and tissue deiodinases [148]. Dysfunction at any stage of this process can compromise gonadal development, reproductive cycling, and embryonic viability [149].

Studies conducted by [150,151] highlight that PAHs can interfere with thyroid function at multiple levels, including hormone synthesis, peripheral conversion of T4 to T3, and signaling via nuclear TH receptors. These interferences result in significant side effects for reproduction, especially during critical windows of larval and juvenile development. Exposure of some species to water-soluble fractions of petroleum containing PAHs resulted in an increase in T4, without a corresponding elevation in T3, suggesting a block in hormone bioconversion. This dysregulation of T3/T4 ratios can negatively affect metamorphosis, gonadal differentiation, and reproductive behavior [150].

Epigenetic modulation of thyroid function has also been documented as a mechanism of disruption. Exposure to BaP and other mixtures containing PAHs has been shown to cause thyroid hyperplasia and alterations in iodine metabolism, interfering with both TH availability and intracellular signaling [84]. These changes, in addition to directly impacting basal metabolism and somatic growth, may compromise cross-signaling between the HPT and HPGL axes, both of which are interdependent for gonadal maturation and sex hormone production [152].

Some PAH exposure increases free T3 levels while reducing circulating sex steroids in marine fish. This isolated elevation of T3 may represent a compensatory attempt by the organism to maintain energy or maturational metabolism under chemical stress, but it may also aggravate the dysregulation of other hormonal axes, since T3 modulates the expression of steroidogenic and gonadotropic genes [151].

Dysregulation of the thyroid cascade can compromise the timing of reproduction, gamete quality, and larval survival, affecting long-term population persistence, as evidenced by [153], who observed that chronic exposure to PAHs combined with warmer winter temperatures disrupted the reproductive phenology of *Platichthys flesus*, delaying gonadal maturation and potentially extending the spawning period, with consequences for recruitment timing in estuarine environments.

PAHs disrupt the thyroid physiology of marine fish through various mechanisms: alteration in hormone synthesis and conversion, interference in iodine uptake, inhibition of deiodinases, activation of nuclear receptors, and epigenetic modulation of gene expression [111]. These changes directly impact reproduction through effects on the HPT axis and its connections with other neuroendocrine axes, especially HPGL. Considering the importance of THs for the life cycle of marine fish, thyroid dysfunction caused by PAHs should be seen as a first-order reproductive risk, with the potential to compromise both individual reproduction and the ecological resilience of exposed populations [152].

### 5.7. Histological Changes in Gonads

Histological changes in the gonads of marine fish exposed to PAHs represent an unequivocal marker of the reproductive toxicity of these compounds. While the molecular and hormonal effects may be transient or compensated by regulatory mechanisms, histological damage is often irreversible and reflects long-term structural and functional dysfunctions, with a direct impact on fertility and population reproduction [154].

Recent studies demonstrate that exposure to PAHs is associated with follicular atresia, delayed gonadal development, changes in spermatogenesis, testicular necrosis, and disorganization of the germinal epithelium [115,155]. These changes have been consistently observed both in controlled experimental conditions and in marine environments contaminated by oil and its derivatives [107].

It is worth noting that histological damage is not homogeneous between the sexes. While females tend to present follicular atresia and ovarian immaturity, males often exhibit germinal necrosis, disorganization of the seminiferous tubules, and reduced sperm production [152]. Such alterations reflect differences in reproductive physiology and sensitivity to disruptive hormonal pathways, especially those mediated by estrogens and androgens.

The study by [94] also pointed out that exposure to BaP caused delayed sexual maturation and gonadal deformations in marine medaka, with clear implications for sex ratio and fertilization rates. This confirms that histological dysfunction of the gonads is not only a consequence of toxicity but also an important causal link between chemical exposure and reproductive collapse [156].

Finally, it is important to consider that histological alterations can be used as reliable biomarkers in environmental biomonitoring, especially when associated with other functional and molecular markers [109]. The presence of anomalous gonadal structures, such as ovotestes or the absence of maturing gametocytes, is indicative of chronic exposure and suggests potential transgenerational disruption, since the integrity of the gonads is essential to produce viable embryos [141].

### 5.8. Impacts on Sex Ratio and Intersex Incidence

Exposure to PAHs has been associated with significant changes in sex ratio and increased incidence of intersex in marine fish, demonstrating their ability to profoundly interfere with sex determination and differentiation [108]. These effects, although sometimes subtle, have the potential to destabilize population structure, since the balance between males and females is essential for maintaining fertility and genetic variability [129].

Disruption in sex ratio occurs, in many cases, through mechanisms related to the modulation of aromatase [94,157], which can cause a delay in the hormonal stimulus required for gonadal development, leading to a disruption in sexual maturity. This indicates that PAHs also affect the timing and speed of sexual maturation, potentially disrupting the synchrony between the sexes and reducing the reproductive efficiency of the group [94].

These changes are not only hormonal or molecular but also have clear morphological consequences. The presence of intersex individuals, fish with testes containing oocytes or ovaries with testicular structures, has been reported in areas contaminated by oil and PAHs, with the formation of oocytes in testes of exposed males [10,129]. Although intersex is more common in freshwater environments, there is growing evidence that marine species are also affected, especially in coastal regions polluted by industrial effluents and oil spills [154,158].

From an ecological perspective, these changes are alarming. Male feminization, detected by the induction of VTG in male organisms, can reduce sperm production, alter sexual behavior, and compromise fertilization. On the other hand, masculinization of females, by suppression of the estrogenic pathway, can lead to incomplete ovulation, spawning failures, or deregulation and infertility [157]. In both cases, the presence of intersex individuals and the alteration of the sex ratio can seriously compromise reproductive dynamics and population resilience [140,156,159].

Furthermore, mate choice is a critical component of sexual selection and an important determinant of individual fitness in fish. To date, no studies have directly investigated the effects of PAHs on mate choice or sexual selection, although evidence from other endocrine-disrupting chemicals indicates that altered sex ratios, feminization, or masculinization can impair courtship behavior, mate recognition, and reproductive success [83]. This gap highlights an important avenue for future research on the ecological impacts of PAHs.

The effects on the sex ratio and occurrence of intersex individuals in marine fish are worrying not only because they compromise individual reproduction but also because they can result in silent population collapses, where fertility gradually deteriorates even in apparently abundant populations [140]. Investigation and monitoring of these phenomena should be a priority in marine ecosystems contaminated by oil and PAHs.

### 5.9. Gonadosomatic Index (GSI)

The gonadosomatic index (GSI), defined as the ratio between the weight of the gonads and the total body weight of the fish, is widely used as a physiological indicator of sexual maturation and reproductive investment in aquatic organisms [160]. In marine fish, this index is particularly informative, as it reflects the seasonal cyclicity of reproduction, hormonal status, and the progression of gametogenesis [161]. Reductions in GSI are often associated with endocrine disruption, gonadal tissue damage, or failures in energy allocation for reproduction and are therefore an integrative marker of reproductive health in the face of environmental stressors such as PAHs [108].

Recent studies have shown that exposure to PAHs can significantly reduce GSI in marine fish, reflecting the interference of these contaminants in gonadal function, such as decreased egg production. Bioaccumulation of PAHs in the ovaries, inhibition of vitellogenesis, and increased follicular atresia are mechanisms associated with the reduction in functional gonadal mass, which together can explain the observed decrease in GSI [54,162].

Field evidence corroborates the experimental findings. In silversides (*Fundulus grandis*) collected in areas impacted by the Deepwater Horizon spill in the Gulf of Mexico, males had GSI approximately 50% lower than those from reference areas one year after the event [163]. Females from these same regions showed underdeveloped oocytes and gonads with lower mass, suggesting that chronic contamination by PAHs may compromise the reproductive investment and reproductive success of exposed populations in a lasting way [108].

The physiological and hormonal mechanisms underlying the reduction of GSI by PAHs are multiple and interrelated. The suppression of sex hormones, such as E2 and 11-KT, is widely documented in marine fish exposed to PAHs [86]. This hormonal decline compromises the progression of gametogenesis, leading to the inhibition of vitellogenesis in females and incomplete spermatogenesis in males, resulting in less developed gonads and, consequently, reduced GSI. Furthermore, inhibition of aromatase, as already discussed, has been consistently observed in fish exposed to PAHs, contributing to hormonal imbalance and impaired gonadal maturation [87].

Histological changes in the gonads, especially follicular atresia, are another critical factor for the decline in GSI. By promoting the degeneration of developing oocytes, PAHs contribute to the loss of functional gonadal mass. The presence of atresia in advanced vitellogenic stages indicates that the oocyte development process was interrupted late, resulting in substantial losses in reproductive biomass [88].

As a consequence of the decrease in GSI, there is not only a decrease in individual fertility but also a decrease in spawning success and population recruitment, especially in species with seasonal reproduction or low fecundity [153]. Therefore, GSI proves to be not only a useful marker for measuring reproductive status but also a sensitive indicator of the sublethal effects of contaminants such as PAHs.

## 6. Cell Signaling Mechanisms Involved in PAHs’ Impacts on Fish Reproduction

As previously described, extensive evidence gathered over decades shows that PAHs can disrupt fish reproductive systems [10]. This disruption primarily results from PAHs’ interference with the endocrine system, as well as their cytotoxic and mutagenic effects on germ cells [164].

PAHs can mimic endogenous estrogen molecules and disrupt estrogenic signaling through both genomic and non-genomic pathways [165,166]. The genomic pathway represents the classical mechanism of estrogen signaling and primarily involves nuclear receptor proteins known as estrogen receptors (ERs), specifically ERα, encoded by the *Esr1* gene, and ERβ, encoded by the *Esr2a* and *Esr2b* genes [167]. In fish, these receptor isoforms play a crucial role in regulating gonadotropin expression and vitellogenesis [139]. Additionally, these nuclear receptors play a critical role in regulating the expression of various genes related to reproduction and hormonal balance [168]. In contrast, the non-genomic pathways engage intracellular signaling cascades and membrane proteins, such as the G protein-coupled estrogen receptor 1 (*Gper1*), which is crucial for regulating the non-genomic estrogen pathway in aquatic organisms [169].

**Figure 3:**
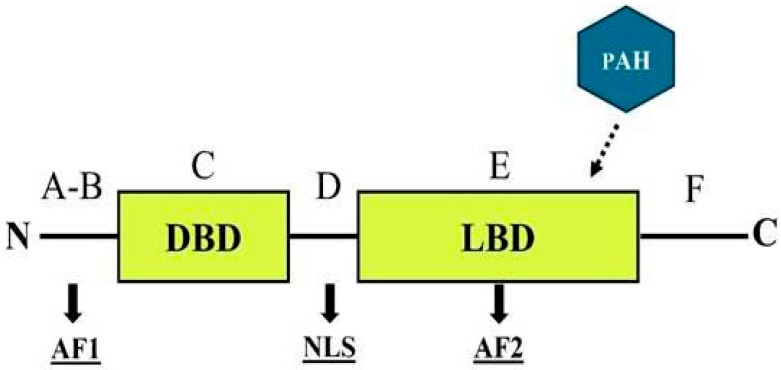
General structure of a nuclear estrogen receptor, emphasizing the most probable interaction site for PAHs. DBD = DNA binding domain; LBD = ligand binding domain; AF1-2: = transcriptional activation function 1 and 2; NLS = nuclear localization signal.

ERs are part of a superfamily of nuclear receptors that specifically bind to small molecules known as ligands, including steroids and thyroid hormones, and are characterized by a distinct domain structure [170]. The ER structure comprises six functionally distinct regions, labeled A through F (Figure 3), that can interact with environmental contaminants in various ways [171,172].

The A–B regions contain a transcriptional activation function (AF-1) located in the N-terminal domain, which exhibits significant variability across species. The C region encompasses the DNA-binding domain, which is highly conserved among species, with a similarity level of 97%. The D region acts as a flexible hinge and contains a nuclear localization signal (NLS). The E region includes the ligand-binding domain (LBD), which harbors a ligand-dependent transcriptional activation function (AF-2). This domain is crucial for most receptor functions, including hormone binding, dimerization, and interactions with coregulators. Finally, the F region extends from AF-2 to the C-terminus of the receptor [170,172]. Although its specific function remains poorly understood, studies suggest that the ER F region may play a role in modulating LBD activity [173,174].

In the absence of ligands, ERs are primarily located in the cytoplasm, where they are associated with heat shock inhibitory proteins [170]. When ligands such as 17β-estradiol are present, the inhibitory complexes disassemble, enabling the ligand to attach to the ER. This binding triggers conformational changes that lead to dimerization, followed by the translocation of the receptor to the nucleus, where it interacts with specific DNA regions in the promoters of target genes, known as estrogen response elements (*EREs*) [175].

PAHs can act as endocrine disruptors by interacting with estrogen receptors, primarily within the ligand-binding domain (LBD), thereby activating estrogen response mechanisms [176]. Although research has long established that PAHs and their metabolites can interact with estrogen receptors [177], their specific effects on estrogen signaling vary depending on the PAH type, the estrogen receptor isoforms involved, and whether exposure occurs as a single compound or a mixture of PAHs [10].

A recent study conducted a virtual screening of 72 PAHs and identified a positive correlation between the number of aromatic rings in these compounds and their binding affinity to key proteins involved in endocrine regulation in zebrafish (*Danio rerio*) [139]. The findings specifically indicated that as the number of aromatic rings in each PAH increased, its binding affinity to the targeted proteins also strengthened. In this context, while E2 (estradiol) and EE2 (17α-ethynylestradiol) were used as true ligands for Erα, comparisons with the 72 PAHs revealed that benzo(g)chrysene, benzo(k)fluoranthene, benzo(e)pyrene, benzo(a)anthracene, and benzo(c)phenanthrene exhibit high binding affinity scores ranging from −11.5 to −9.6 kcal/mol. Notably, all of these PAHs demonstrated 2D and 3D similarity scores exceeding 0.5 and 0.7, respectively. Among them, benzo(g)chrysene displayed the highest 3D similarity to EE2, with an almost perfect structural overlap [139].

Estrogenic responses can also be mediated through non-genomic pathways, wherein estrogen binds to membrane or cytoplasmic receptors, such as G-protein-coupled estrogen receptor 1 (*Gper1*), initiating a rapid intracellular signaling cascade [178]. However, beyond endogenous estrogens, various environmental pollutants have been shown to interact with *Gper1*, potentially functioning as endocrine disruptors [178]. For instance, exposure to a mixture of 16 PAHs resulted in the downregulation of *Esr1* and *Gper1* in the human granulosa cell line (HGrC1). These genes are involved in genomic and non-genomic estrogenic pathways, respectively, and their suppression was associated with a reduction in estradiol secretion [179]. Conversely, no significant alterations in *Gper1* expression were observed in primary hepatocytes of *O. niloticus* following exposure to a commercial EPA PAH mixture [180]. In this context, a 2018 review highlighted the lack of substantial evidence supporting the activation of non-genomic estrogenic signaling by PAHs [181]. However, compared to genomic pathways, research on the effects of PAHs on non-genomic estrogenic mechanisms, particularly in fish models, remains limited, thereby constraining a comprehensive understanding of their potential impact.

Compared to female fish, the reproductive toxicity of PAHs in male fish remains less well understood. Although some studies suggest that PAH exposure can disrupt testosterone levels, the effects reported in the literature vary depending on the specific PAH compounds and the fish species examined. Despite the scarcity of studies elucidating these mechanisms in marine species, the literature provides several findings in freshwater fish, which help illustrate how PAHs can affect male endocrine regulation. For instance, decreased testosterone levels have been observed in *Danio rerio* exposed to phenanthrene at concentrations of 5 and 50 nmol/L [133]. Similar effects were observed in the turbot (*Scophthalmus maximus*) exposed to PAHs extracted from Prestige fuel oil, as well as in the white-throated slug (*Catostomus commersonii*) from a river site polluted with PAHs [182]. Conversely, PAH exposure has been shown to enhance testosterone production in goldfish and rainbow trout by stimulating testicular steroidogenesis [183]. These contrasting findings highlight the complexity of PAH-induced endocrine disruption and underscore the need for further research to elucidate species-specific and compound-specific effects.

Similar to estrogen receptors, the structure of androgen receptors (AR) is composed of three main domains: an N-terminal hypervariable transcriptional activation domain (TAD), a central highly conserved DNA-binding domain (DBD), and a C-terminal ligand-binding domain (LBD) [184]. The LBD also plays a crucial role, as it is where the modulation of receptor activity takes place [139]. Due to the high conservation of these protein domains between fish and mammals, many antiandrogenic effects caused by environmental contaminants are linked to reproductive dysfunctions that can impact population size, such as decreased sperm quality in various vertebrates [142].

Androgen receptors perform their functions in a manner very similar to that previously described for estrogen receptors. In the absence of ligands, AR remains in the cytoplasm, associated with chaperone proteins, primarily heat shock proteins [185]. When an androgen binds to AR, it induces a conformational change, leading to the dissociation of these chaperone proteins and the exposure of the NLS region. The androgen/AR complex then translocates to the nucleus, dimerizing and binding to specific promoter regions known as androgen response elements (AREs) within classical target genes. Thus, it modulates gene transcription [186]. The transcriptional activity of the androgen-bound AR is also influenced by specific proteins called coregulators. These proteins bind to the activated AR in a ligand-dependent manner, either enhancing its ability to transactivate target genes as coactivators or repressing it as corepressors [187,188].

A molecular docking study was conducted to assess the binding affinity of several PAHs—cyclopenta[cd]pyrene, benzo(b)chrysene, dibenz(a,h)anthracene, benzo(k)fluoranthene, and dibenzo(a,e)pyrene—toward the androgen receptors of *D. rerio*, using testosterone and dihydrotestosterone (DHT) as endogenous ligands. The results revealed that these PAHs exhibited significant 2D and 3D structural similarities to the endogenous ligands, with binding affinities ranging from −9.7 to −8.8 kcal/mol [139]. Based on the information provided, it can be inferred that these PAHs may interact with and modulate the activity of the androgen receptor. These findings suggest that the tested PAHs may interact with and potentially modulate androgen receptor activity. This is particularly concerning given the androgen receptor’s role in regulating gene expression associated with reproductive development and responding to androgenic molecules such as testosterone in mammals, as well as 11-ketotestosterone and 5α-dihydrotestosterone (DHT) in fish [142].

In summary, the documented impacts of environmentally relevant PAHs on fish reproduction necessitate further investigation, particularly concerning their interference with reproductive mechanisms in male and female fish through non-genomic pathways. Additionally, considering the variability in effects across species and the diverse origins of PAHs explored in prior studies, it is imperative that future research enhance ecological relevance by evaluating regionally significant PAHs, both individually and in mixtures, in key native species within their respective ecosystems.

## 7. Limitations and Challenges of Current Methodologies for Biomonitoring of Marine Ecosystems Threatened by PAHs

Biomonitoring is a useful tool for assessing the impact of PAHs on marine ecosystems. Although several biomonitoring approaches have been developed to assess the environmental and biological impacts of PAHs, these methodologies face several limitations and challenges that hinder their effectiveness. The selection of the exposure method, species, and developmental stages, as well as the duration of the study and the biomarkers of exposure or effects, can directly influence the conclusions of the research [189]. PAHs can induce a range of sublethal effects, such as oxidative stress, DNA damage, and endocrine disruption [190]. Laboratory studies often report stronger or more conclusive effects than field and active biomonitoring studies, likely because they are conducted under controlled conditions, usually with higher contaminant concentrations and reduced interference from other environmental variables or chemicals [120]. Therefore, these elements must be carefully chosen based on the specific questions that the researchers intend to investigate.

One of the main challenges in biomonitoring is the selection of suitable species for the assessment of the effects of PAHs. Marine species have varied sensitivities and can develop metabolic adaptations to stressors that make it difficult to detect the adverse effects caused by PAHs [189]. Mollusks and fish are frequently used due to their capacity to accumulate this contaminant, and the responses to exposure vary significantly according to species, sex, age, and physiological conditions [191].

The use of early life stages of marine organisms for toxicity assessment is scarce, although they have advantages over adults and juveniles [191]. Early life stages are critical for the health and recruitment of marine populations, offering advantages for risk assessment in the field. Assessing biomarkers in organisms of only one sex may lead to biased results, since sex differences can increase or decrease the susceptibility of organisms, in addition to presenting physiological differences [192]. Variability in biological models can make it difficult to interpret biomonitoring data and generalize findings across ecosystems.

Biomonitoring of PAH-contaminated environments faces challenges in analytical issues. Detection and quantification in different matrices require the use of advanced analytical techniques and equipment, such as gas chromatography coupled with mass spectrometry (GC-MS) or high-performance liquid chromatography-ultraviolet (HPLC-UV) [193]. One of the main obstacles is the sensitivity of analytical instruments, especially when it comes to monitoring low concentrations in large marine areas. Furthermore, the complexity of the matrices present in biological samples can interfere with detection, resulting in possible underestimations or overestimations of contamination levels [194]. Even with advances in techniques, the sample collection and preparation process is laborious, costs are high, with prolonged analysis time and the need for specialists for the analyses [195]. In addition, PAH monitoring in marine environments is challenged by temporal and spatial variability, which can further complicate the interpretation and generalization of biomonitoring data [196].

Biomonitoring of PAHs is also challenged by the presence of other contaminants and the impacts of climate change [197]. Climate change variables can influence the fate of contaminants throughout marine ecosystems, and the interaction between toxicants and physical stressors can affect the response of biomarkers. Ocean acidification and rising temperatures can interact with PAHs and exacerbate their negative impacts [198]. Biomonitoring methodologies must consider and adapt to the synergistic effects of climate change and marine pollution [199].

Future research should prioritize improving the robustness, sensitivity, and applicability of biomonitoring approaches, aiming to more effectively protect marine ecosystems from contamination by PAHs. To overcome current limitations, it is essential to adopt a multidisciplinary approach that integrates advanced technologies, careful selection of biomarkers, and consideration of the impacts of climate change. In addition, it is essential to intensify efforts to assess the effects of contamination, considering the wide variety of contaminants present in aquatic ecosystems. In this context, the development of non-targeted methods capable of covering the great diversity of pollutants potentially present in these environments becomes an urgent need.

## 8. Patents and Research Trends on the Detection of PAHs and Its Impacts in Aquatic Ecosystems

Accurately estimating the total PAH load entering aquatic environments remains a significant challenge [200]. Nevertheless, assessments of PAH sources and their ecological effects have been documented since the 1980s, and the presence of these contaminants in industrial and municipal effluents, as well as in adjacent environments, has been recognized since the 1970s [201,202]. Despite this long-standing awareness, identifying the specific sources of PAHs to effectively prevent environmental contamination remains a major limitation, largely due to their typically low concentrations in aquatic ecosystems [203].

Since the United States Environmental Protection Agency (U.S. EPA) has listed the 16 PAHs for monitoring [204], various methodologies have been used to measure concentrations in water, namely, gravimetry, chromatography, fluorescence, and spectrometric analysis. Gas chromatography (GC), mass spectrometry, and fluorescence spectrometry in special have been providing reliable results in many cases, and fluorescence methods for detection of petrogenic PAHs can generate fast and cost-effective results [205]. However, conventional methods may supply the demand of analyses nowadays, and market analysis for PAHs indicates a growing demand for testing services due to increasing environmental regulations and concerns about their potential health risks [206]. As a result, an increase in knowledge and technological improvements has occurred in recent years, showing new monitoring strategies and detection of PAHs.

In this section, an overview of technologies on various patent databases will be presented. To shortlist potentially relevant patents, the search was narrowed by submitting the keywords, i.e., “polycyclic aromatic hydrocarbons, PAH, detection, aquatic”. The search was conducted on WIPO (World Intellectual Property Organization) and EspaceNet (EPO—European Patent Office) and followed by screening patents of interest. A total of 25 patents were selected as related to technologies to detect PAHs in aquatic environments.

During this search it was noticeable that most of the collections related to PAHs in terms of environmental contamination and biotechnology are related to bioremediation of hydrocarbon and food analysis technologies. At first search in those databases, almost a thousand patents were found, but only 25 were relevant (Table 3) at detection and aquatic environment monitoring. As PAHs are naturally found, present technologies here may collaborate to eventually map possible non-natural origins of these contaminants.

The complete analysis of the collected patent documents was conducted and showed technological advancements in the field of analysis, detection, and monitoring of PAHs in the environment. The results of patent filings showed similarities with five broad categories as follows:

- Technologies that may assist conventional methods (i.e., GC, HPLC, spectrometry) for better sensibility, accuracy, or low cost;
- Protocol or apparatus with some advantage over the conventional ones;
- Innovative new apparatus;
- Methodology to analyze, prevent, and monitor environmental risks by PAHs and even other contaminant accidents;
- Bioindicators are serving to detect and monitor PAH contamination.

### 8.1. Technologies That May Assist Analytical Methods

Reinforcing the challenges found in conventional analytical methods, such as low selectivity leading to the non-detection of isomers or PAHs already incorporated into the sample, the technology reported by the patent US11029303B2 [209] proposed the use of two specific antibodies targeting two PAHs. This patented technology facilitates the production of these specific biological markers, particularly for methylated phenanthrenes, which are major PAHs in petroleum. These markers enhance fluorescence analyses, improving both precision and selectivity.

Some samples, due to the low concentration of pollutants, may have their results further compromised or even remain undetected. To increase sensitivity, the patent CN115078579A [211] developed a technique utilizing naphthyl-modified magnetic ferroferric oxide to assist in PAH detection.

### 8.2. Protocol or Apparatus with Some Advantage over the Conventional Ones

The determination of PAHs in aquatic samples is often performed using gas chromatography-mass spectrometry (GC-MS). Conventional methods are time-consuming and prone to false positives due to matrix interferences [231]. To avoid such problems, the patent CN102854280A [208] proposes a protocol that makes use of Gas Chromatography-Triple Quadrupole Tandem Mass Spectrometry (TSQ Quantum GC), allowing the detection of 16 PAHs at a concentration of 10 μg/kg.

Common instrumental detection methods include liquid chromatography (LC), gas chromatography-mass spectrometry (GC-MS), and high-performance liquid chromatography (HPLC). The HJ 478-2009 method, a standard for PAH detection in water samples, involves liquid-liquid extraction and solid-phase extraction. However, it has significant drawbacks, including cumbersome and costly pre-treatment, and its fluorescence detector is not suitable for detecting acenaphthene. To address these limitations, a novel approach described by the patent CN112305116A [215] using CSR-LVSI technology has been developed to optimize PAH detection, significantly improving analytical sensitivity and meeting stricter environmental testing requirements.

Additionally, a molecular probe with environmentally sensitive photoluminescence has been disclosed by patent US11561175B2 [226] as a method for detecting hydrocarbon contamination in samples, representing an innovative alternative in PAH analysis. These methods refer to a technique of embodiment of PAHs in water using DNS and DNS-OH as molecular rotors.

### 8.3. Innovative New Apparatus for Analysis

A novel invention presented by patent WO2016207461A1 [217] is a passive ceramic sampler for measuring water pollution. This device consists of a porous ceramic casing filled with an adsorbent material, allowing for the pre-concentration of pollutants in water to determine its chemical quality. Namely, passive sampling is an integrated method of sample collection that relies on the free flow of pollutants in the environment to the receiving system. The sampling period is determined by the sampling device. The movement of analytes occurs due to differences in chemical potential between the outside and inside of the sampler until steady state or the end of the sampling period is reached.

Another advancement related to passive sampling devices designed by the patent US10551283B2 [225] is for analyzing environmental matrices such as surface water, aquatic sediments, and soil. It addresses the challenges of slow equilibrium in static sediments, and researchers developed a method to manipulate the external depletion layer of the sampler. This involves mechanically disrupting the depletion layer using periodic in situ vibrations, enhancing the accuracy of freely dissolved analyte concentration measurements in sediment porewater.

### 8.4. Methodology to Analyze, Prevent, and Monitor Environmental Risks of Contamination

A critical challenge in oil mining is establishing a reliable method for identifying and calculating ecological risks. The patent CN102062769A [221] proposes a methodology that integrates regional ecological risk assessment with data analysis. This approach considers the characteristics of oil mining pollutants, receptor sensitivity, and other key indicators. It is mainly based on probabilistic modeling rather than direct analytical measurements, as it uses a joint probability distribution function with a double integral method to estimate regional ecological risk status and support environmental risk prevention efforts.

### 8.5. Bioindicators Serving to Detect and Monitor PAH Contamination

One novel approach of patent CN111208270A [222] involves using three spiny fish CYP1 family genes as biomarkers for water pollution detection. This method provides a biological alternative to conventional chemical techniques, offering sensitive detection of PAHs through gene expression responses.

The patent US10871484B2 [223] showed that enzymatic assays have emerged as another promising tool for PAH detection. Unlike traditional methods such as GC-MS and HPLC, enzyme-based assays require less sophisticated instrumentation, making them more accessible and efficient. These assays are widely used in clinical applications for detecting DNA mutations, post-translational protein modifications, and serum content analysis. Furthermore, enzyme assays are compatible with surfactants, making them preferable for PAH extraction when hazardous organic solvents are impractical, costly, or environmentally unsafe.

However, enzyme-based methods also present methodological constraints, since their activity can be affected by temperature and other environmental variables, which may limit their robustness for routine environmental monitoring.

### 8.6. Other Considerations

The research covered in this section presents a diverse range of methodologies and technological innovations aimed at improving PAH detection, environmental monitoring, and pollution risk assessment. From antibody-based fluorescence techniques and molecular probes to advanced passive samplers and enzymatic assays, the field has evolved significantly to address the limitations of conventional methods. These advancements not only enhance analytical accuracy and sensitivity but also contribute to more efficient, cost-effective, and environmentally sustainable monitoring solutions. Given the ongoing need for improved pollution detection and ecological risk management, continued innovation and integration of these emerging technologies will be crucial in shaping future environmental analysis and methodologies.

## 9. Conclusions

PAHs remain a major class of environmental contaminants in marine ecosystems, with proven capacity to disrupt fish reproduction through endocrine interference, genotoxicity, and oxidative damage. Early life stages are particularly vulnerable, emphasizing the ecological relevance of developmental biomarkers in environmental assessments. Despite the growing use of biochemical and molecular biomarkers for monitoring PAH exposure and reproductive effects in fish, significant knowledge gaps persist regarding the long-term and population-level consequences of such exposure.

Furthermore, the bioavailability and bioaccumulative potential of PAHs in aquatic organisms raise concerns not only for ecosystem integrity but also for human health via biomagnification through the food web [46,232]. Also, a critical limitation in addressing PAH contamination lies in the regulatory framework. Current legislation varies considerably across countries and is often restricted to a limited subset of PAHs while neglecting the broader spectrum of compounds with known or suspected toxicity [233]. The detection of PAHs in local biota underscores the urgent need for more inclusive, compound-specific regulatory measures and consistent environmental monitoring [234]. Addressing these regulatory and scientific gaps will be essential for developing effective strategies to mitigate PAH-related risks in marine environments and protect both ecological and human health in the face of ongoing contamination pressures.

From an applied perspective, these findings also have important implications for fisheries and aquaculture. Reproductive impairment, altered sex ratios, and developmental toxicity in fish can directly affect stock recruitment and population sustainability, threatening wild fishery resources. In aquaculture settings, chronic PAH exposure may compromise broodstock fertility, larval survival, and growth performance, ultimately reducing productivity and economic viability. Considering the dependence of coastal communities on both fisheries and aquaculture, addressing PAH contamination is essential not only for ecosystem conservation but also for food security and sustainable resource management.

While substantial progress has been made in understanding the effects of PAHs and related compounds on fish, a few questions remain to be addressed. The findings highlighted in this paper show the exquisite sensitivity of fish in early stages to petrogenic compounds. There is substantial evidence that PAHs can cause reproductive impairment in a variety of fish through their ability to disrupt endocrine function and their cytotoxic and mutagenic effects on germ cells. Therefore, future studies are necessary to unravel the complexity of the toxic effects of PAHs.

## Figures and Tables

**Table 1:** Effects of PAHs on different species of fish.

Species	Common Name	Compound	Observed Effect	Matrix and Concentration	Reference
*Fundulus heteroclitus*	Mummichog	Benzo[a]pyrene	Hepatocellular carcinomas	Filtered seawater; 400 µg/L	[58]
*Chanos chanos*	Milkfish	Anthracene	Oxidative stress Neurotoxicity	Seawater; 0.188 mg/L	[59]
*Chanos chanos*	Milkfish	Benzo[a]pyrene	Increased catalase and lipid peroxidation; inhibition of acetylcholinesterase	Seawater; 0.031 mg/L	[59]
*Pomatoschistus microps*	Common goby	Pyrene	Inhibition of acetylcholinesterase	Artificial salt water; 20 µg/L	[60]
*Cynoscion nebulosus*	Spotted seatrout	Total PAHs (oil spill)	Reduction of lymphocytes and increase of splenic melano-macrophages; increased activity of EROD	Seawater	[61]
*Fundulus grandis*	Gulf killifish	Total PAHs (oil spill)	Reduction of lymphocytes and inflammatory response	Seawater	[61]
*Pagrus major*	Red seabream	Benzo[a]pyrene	Increased EROD activity; increased cortisol level; and liver damage	Seawater; 4 and 8 µg/L	[62]
*Melanogrammus aeglefinus*	Haddock	Dispersed crude oil (parental and alkylated PAHs)	Cardiotoxicity and craniofacial malformation in embryos	Seawater; >0.7 µg/L	[63]
*Mugilogobius chulae*	Chulae’s goby	Benzo[a]pyrene	Inhibition of genes associated with lipid metabolism	Seawater; 6 µg/L	[64]
*Boreogadus saida*	Arctic cod	Total PAHs	PAH bioaccumulation	Biological tissue; 36 to 128 ng/g	[65]
*Girella punctata*	Largescale blackfish	Benzo[a]anthracene	Spinal deformity; liver damage	Seawater: 10 ng/g	[66]
*Pagrus major*	Red seabream	Phenanthrene	Increased activity of SOD and EROD in serum and liver	Seawater; 20 µg/L	[67]
*Oncorhynchus tshawytscha*	Chinook salmon	Petroleum-derived total PAHs light molecular weight (LMW) and heavy molecular weight (HMW)	Immune suppression of phagocytic activity and lymphocyte proliferation	Sediment (LMW: 120 ± 120 mg/g; HMW: 21 ± 15 mg/g)	[68]
Teleost fish species (various)	-	Petroleum-derived total PAHs	Developmental defects in embryos and larvae	Sediment and seawater (unspecified concentrations)	[69]
Teleost fish species (various)	-	Total PAHs	Physiological effects (e.g., metabolic, physiological, and reproductive impacts)	Sediment and seawater (unspecified concentrations)	[70]
*Oryzias melastigma*	Marine medaka	Phenanthrene	Teratogenic effects on early development	Seawater; 0.1–0.3 mg/L	[71]

Notes: only statistically significant effects reported in the original studies are presented.

**Table 2:** Summary of studies on the reproductive effects of PAHs in marine fish.

Species	Common Name	Compound	Exposure Conditions	Reproductive Effects	Reference
*Epinephelus marginatus*	Dusky grouper	Phenanthrene	Waterborne exposure of juveniles for 96 h to 0.1 and 1 mg/L	Dysfunctions in steroidogenesis; reduction of 11-KT in vitro; impairment in sexual inversion and gonadal maturity	[86]
*Oryzias melastigma*	Marine medaka	Phenanthrene	Waterborne exposure of juveniles for 80 days to 0.06, 0.6, 6, and 60 g/L	Reduction of vitellogenic oocytes; downregulation of GnRH, FSH, LH, CYP19A, ER, and VTG; reduced hatchability in embryos	[87]
*Solea solea*	Common sole	Benzo[a]pyrene	Waterborne exposure of adult females for 15 days to 21 and 50 µg/L	Hepatic and ovarian accumulation of BaP; oocyte atresia; reduced gonadosomatic index; decreased ER-α; increased hepatic aromatase	[88]
*Gadus morhua*	Atlantic cod	Mixture of PAHs (Benzo[a]pyrene, Dibenzothiophene; Fluorene, Naphthalene, Phenanthrene, and Pyrene)	Intraperitoneal exposure of juvenile females for 14 days to 40 and 800 µg/kg	Reduction of dopamine and metabolites; alteration of dopaminergic and estrogenic gene expression; decrease in ER-α and CYP19a	[89]
*Paralichthys lethostigma*	Southern flounder	Oil (sediment + Macondo MC252 oil)	Water-benthic exposure of juveniles for 32 days to 0, 0.7, 8, 54, 127, and 395 mg/kg	CYP1a induction in liver; increased mortality; reduced growth with high concentrations of PAHs	[90]
*Oryzias melastigma*	Marine medaka	Phenanthrene; Phenanthrene attached to microplastics	Waterborne exposure of adult females for 60 days to 50 μg/L Phenanthrene and 2, 20, and 200 μg/L of microplastics	Bioaccumulation of Phenanthrene in the ovaries; dysfunctions in the HPG axis; reduction of VTG level; inhibition of ovarian maturity; downregulation of LHβ, FSHβ, CYP17, and CYP19a	[91]
*Liza klunzingeri*	Klunzinger’s mullet	Benzo[a]pyrene	Culture of ovarian and brain cells of adult females; exposure of the cells for 48 h to 1 × 10^−6^, 2 × 10^−6^ and 3 × 10^−6^ mol/L	Aromatase inhibition; reduction in E2 production; dysfunctions in estrogen biosynthesis	[92]
*Gadus morhua*	Atlantic cod	Benzo[a]pyrene; Benzo[a]pyrene + EE2	Ex vivo exposure of liver slices of juveniles to 0.1, 1, and 10 μM, alone and mixed with EE2, for 48 h	Upregulation of the CYP and AhR genes; downregulation of VTG and ZNF; exposure to the mixture maintained the expression profiles of the individual compounds, but with attenuation of the estrogenic effects of EE2	[93]
*Oryzias melastigma*	Marine medaka	Benzo[a]pyrene	Waterborne exposure of embryos, juveniles and adults for 142 days to 2, 20, and 200 µg/L	Delayed hatching; altered sex ratio; downregulation of ERα, CYP19a, and VTG1; increased androgen receptors	[94]
*Ancylopsetta dilecta*, *Cyclopsetta chittendeni*, *C. fimbriata*, *Monolene sessilicauda*, *Syacium micrurum*, *S. papillosum*, *S. gunteri* and *Trichopsetta ventralis*	Three-spot flounder; Mexican flounder; Fringed flounder; Deepwater sole; Channel flounder; Dusky flounder; Shoal flounder; and Sargassum flounder (respectively)	-	Field study with animals collected in two regions of the Gulf of Mexico	VTG induction in males; association with metals and biliary PAH metabolites; evidence of feminization and inhibited spermatogenesis	[95]
*Sebastes schlegelii*	Black rockfish	Benzo[a]pyrene	Intraperitoneal exposure of adults for 48 h to 2, 20, and 200 μg/g body weight	CYP1A induction; presence of VTG and ZRP in immature males and females; possible endocrine dysfunction	[96]
*Boreogadus saida*	Polar cod	Benzo[a]pyrene; Benzo[a]pyrene + EE2	Ex vivo exposure of liver slices of adult females to 0.1, 1 and 10 μM, alone and mixed with 5 nM EE2, for 72 h	CYP1a induction; antiestrogenic effects with co-exposure (reduction of ESR1, VTG, and ZP); activation of AhR	[97]
*Carcharodon carcharias*	Great white shark	-	Field collection of juveniles and adults from South African coast; use of skin biopsy	CYP1A induction; presence of VTG and ZRP in young males and females	[98]
*Oncorhynchus kisutch*	Coho salmon	Oil WAF	Waterborne exposure of juveniles for 96 h to 100, 320, and 1000 mg/L	Induction of CYP1a and AhR; disruption of estrogenic genes such as VTG and CYP19	[99]
*Oryzias melastigma*	Marine medaka	Oil WAF	Waterborne exposure of embryos for 7 days to 0.5, 5, 50, and 500 μg/L, with subsequent creation of organisms until the adult phase (130 dpf)	Decreased GSI, E2, and VTG; increased T, ARα, Arβ, and CYP19b; VTG promoter methylation in adult phase	[22]
*Boreogadus saida*	Polar cod	Crude oil	Waterborne exposure of adults for 131 days to 13 μg/L, with food restriction during spawning; the concentration at the end of the experiment was 0.09 μg/L	Alteration in the expression of VTGα, ER1, ZP2, and ZP3; early spawning; alteration in gamete quality, independent of food restriction	[100]
*Boreogadus saida*	Polar cod	Crude oil	Dietary exposure of adults for 217 days prior to spawning to 0.11, 0.57, and 1.14 μg crude oil/g fish/day	Induction of EROD; alteration of sperm motility	[101]
*Dicentrarchus labrax*	European seabass	Sediments enriched with Phenanthrene and Benzo[b]fluoranthene	Trophic exposure of juveniles for 28 days to contaminated sediment (250–800 ng/g)	PHE induced oxidative stress and CYP activation; BbF compromised metabolic defenses; the mixtures caused liver injury associated with the AhR pathway	[12]
*Melanogrammus aeglefinus*	Haddock	Extracts of water produced from oil (2-ring PAHs); crude oil (3-ring PAHs); and heavy pyrogenic PAHs (4/5/6-ring PAHs)	Trophic exposure of juveniles for 67 days to 0.31 PAH/kg fish/day of water produced from oil to 0.45 PAH/kg fish/day of crude oil, and to 0.65 PAH/kg fish/day of pyrogenic PAHs, followed by a two-month recovery	Heavier PAHs induced CYP1a and AhR expression, increased CYP activity and biliary metabolites; exposure to crude oil showed CYP1a induction	[102]
*Sparus aurata*	Gilthead seabream	Phenanthrene, Benzo[a]pyrene, and Benzo[b]fluoranthene	In vitro exposure of hepatocytes extracted from adults, to isolated (0.1, 1, 10, and 50 μM) and mixed (1:1, 1:2, and 2:1) PAHs, for 24 and 48 h	Benzo[a]pyrene induced *Cyp1A1* gene and protein expression by increasing its activity; mixtures containing Phenanthrene and Benzo[a]pyrene further increased CYP1A1 mRNA levels	[103]
*Gobionotothen gibberifrons*, *Notothenia rossii*, *Chaenocephalus aceratus* and *Champsocephalus gunnari*	Humped rockcod; Marbled rockcod; Blackfin icefish; and Mackerel icefish (respectively)	Benzo[a]pyrene	In vitro exposure of S9 liver fractions from adults, to 0.5, 1 and 2 μM, for 0, 10, 20, 30, and 60 min	Hepatic EROD activity was highest in *C. gunnari* and *C. aceratus*, and slightly lower in *G. gibberifrons* and *N. rossii*	[104]
*Mugil cephalus*	Flathead grey mullet	-	Field collection of adults from western coast of the Black Sea, Turkey	EROD activities and CYP1A levels were highly elevated, correlating with the high levels of PAHs detected in the liver of the animals	[105]
*Lutjanus campechanus* and *Balistes capriscus*	Red snapper and Grey triggerfish, respectively	-	Field collection of adults from north-central Gulf of Mexico	High activities of Benzo[a]pyrene hydroxylase and EROD, which was higher in *B. capriscus*	[106]
*Sciades herzbergii*	Pemecou sea catfish	-	Field collection of adults from an estuary of the Amazon Equatorial Coast contaminated with PAHs	Histological changes in the gonads (melanomacrophages in the ovaries and testes; atretic oocytes and cytoplasmic retraction in the ovaries)	[107]
*Fundulus grandis*	Gulf killifish	-	Field collection of adults from the Gulf of Mexico after the Deepwater Horizon explosion	Higher sex ratio of females; lower GSI and testicular germinal epithelium in males	[108]
*Hippocampus erectus*	Lined seahorse	Benzo[a]pyrene	Waterborne exposure of adults for 7 days to 0.5, 5, and 50 μg/L	Concentration-dependent damage to ovarian, testicular and brood pouch tissue; differential expression of genes related to CYP pathways	[109]
*Acanthopagrus arabicus*	Arabian seabream	Phenanthrene	Intraperitoneal exposure of adults for 21 days to 2, 20 and 40 pg/g of body weight	T3 and T4 levels decreased dose-dependently until day 7 and then increased until the end of the experiment; decreased thyroid follicle epithelial thickness and increased follicle diameter were also observed	[110]
*Liza abu*	Abu mullet	Benzo[a]pyrene	Intraperitoneal exposure of adults to 2, 10, and 25 mg/kg of body weight; samples were collected 1, 2, 4, 7, and 14 days after injection	Decrease in plasma levels of T3 and T4 and increase in TSH concentration	[111]
*Liza klunzingeri*	Klunzinger’s mullet	Naphthalene	Intraperitoneal exposure of adult females for 3 and 72 h to 50 mg/ kg of body weight	Decrease in T4 levels at both times; T3 decreased only after 72 h	[112]

Notes: 11-KT = 11-ketotestosterone; CYP17 = cytochrome P450 family 17 gene; CYP19a = cytochrome P450 family 19 subfamily A; dpf = days post fertilization; EE2 = 17α-ethinylestradiol; ER-α = estrogen receptor alpha; EROD = ethoxyresorufin-O-deethylase; FSHβ = follicle-stimulating hormone beta subunit; GnRH = gonadotropin-releasing hormone; HPG = hypothalamic-pituitary-gonad; LHβ = luteinizing hormone beta subunit; PAH = polycyclic aromatic hydrocarbons; VTG = vitellogenin; WAF = water accommodated fraction; ZP = zona pellucida; ZRP = zona radiata protein. Only statistically significant effects reported in the original studies are presented.

**Table 3:** Selected patent survey about technologies to detect PAHs.

**Patent Publication Number**	**Patent Content**	**Publication Date (dd/mm/yyyy)**	**Applicant Country**	**Reference**
CN118604201A	Method for detecting multiple PAHs in aquatic products and application thereof	06.09.2024	China	[207]
CN102854280A	Secondary mass spectrometry method for detecting 16 kinds of PAHs in aquatic products	02.01.2013	China	[208]
US11029303B2	Antibody compositions that specifically bind to PAHs	08.06.2021	United States of America	[209]
US2015377752A1	Determination of PAH in water using nanoporous material prepared from waste avian eggshell	31.12.2015	United States of America	[210]
CN115078579A	Application of naphthyl-modified magnetic ferroferric oxide nanoextraction material in enrichment and detection of PAHs	20.09.2022	China	[211]
RU2589897C1	Method of determining general and PAHs in components of ecosystem	10.07.2015	Russia	[212]
KR101199440B1	PAH exposure-responsive genes in *Scleronephthya gracillimum* and the method for diagnosing the coastal environment pollution using the same	09.11.2012	Korea	[213]
CN115561338A	Method for determining PAH pollution in shellfish by adopting gas chromatography-mass spectrometry	03.01.2023	China	[214]
CN112305116A	Method for determining ultra-trace PAH in water	02.02.2021	China	[215]
CN118063840A	Preparation method and application of magnetic metal-organic framework composite material MUiO-BDA	24.05.2024	China	[216]
WO2016207461A1	Passive ceramic sampler for measuring water contamination	29.12.2016	Spain	[217]
CN118913795A	Chemical analyzer for high-selectivity detection of PAH in the environment	08.11.2024	China	[218]
AU2020101615A4	A method for source apportionment of PAHs in roadway sediments coupled with transport and transformation processes	10.09.2010	Australia	[219]
CN116159342A	DGT device for PAH monitoring and application thereof	26.05.2023	China	[220]
CN102062769A	Method for recognizing ecological risks and calculating value at risk for land oil exploitation	18.05.2011	China	[221]
CN111208270A	Application of the three-spined stickleback CYP1 family gene in the preparation of a water body pollution detection biomarker and the detection method thereof	29.05.2020	China	[222]
US10871484B2	Enzymatic method for detecting PAHs	22.12.2020	United States of America	[223]
AU2017200771A1	Identification of PAHs	23.08.2008	Australia	[224]
US10551283B2	Actively shaken in-situ passive sampling device	04.02.2020	United States of America	[225]
US11561175B2	Detection of hydrocarbon contamination in soil and water	24.01.2023	Germany	[226]
RU2017109544A	Method for determining the total content of monocyclic aromatic hydrocarbons in water	21.09.2018	Russia	[227]
US20140088209	Extraction of harmful compounds from materials containing such harmful compounds	19.09.2013	United States of America	[228]
US20140154723	Method of detecting the presence of PAHs	05.06.2014	United States of America	[229]
WO2016196638	PAH antibodies and uses thereof	08.06.2016	United States of America	[230]
